# Targeting WDR12 Unleashes T‐Cell‐Mediated Antitumor Activity in Melanoma by Destabilizing CD276

**DOI:** 10.1002/advs.76255

**Published:** 2026-06-27

**Authors:** Jie Pan, Ruimin Chang, Meng Zhang, Qian Dong, Guanxiong Zhang, Xiang Chen, Mingliang Chen, Lixia Lu, Xiaowei Liang, Yeye Guo, Juan Su

**Affiliations:** ^1^ The Department of Dermatology Xiangya Hospital Central South University Changsha China; ^2^ National Engineering Research Center of Personalized Diagnostic and Therapeutic Technology Changsha China; ^3^ Furong Labratory Changsha Hunan China; ^4^ National Clinical Research Center for Geriatric Disorders Xiangya Hospital Changsha China; ^5^ Hunan Key Laboratory of Skin Cancer and Psoriasis Xiangya Hospital Changsha China; ^6^ Hunan Engineering Research Center of Skin Health and Disease Xiangya Hospital Changsha China; ^7^ The Department of Thoracic Surgery Xiangya Hospital Central South University Changsha Hunan China; ^8^ State Key Laboratory of Proteomics Beijing Proteome Research Center National Center for Protein Sciences ‐ Beijing Beijing Institute of Lifeomics Beijing China

**Keywords:** CD276(B7‐H3), immunotherapy resistance, melanoma immunotherapy

## Abstract

Melanoma is the deadliest skin cancer, and despite the success of immune checkpoint blockade, a substantial fraction of patients fail to respond. Tumors can evade cytotoxic lymphocytes by upregulating multiple inhibitory checkpoints. Here we identify WDR12 as a determinant of immunotherapy resistance: WDR12 expression is elevated in nonresponders, and its genetic inhibition increases intratumoral CD8+ T‐cell infiltration and enhances cytotoxic function. Mechanistically, WDR12, in cooperation with the chaperonin subunit CCT7, stabilizes the immune checkpoint CD276 (B7‐H3) on tumor cells, thereby suppressing T‐cell activity and promoting immune escape. To translate these findings, we identify SU14813 as a small‐molecule WDR12 inhibitor that binds WDR12 with high specificity, reduces CD276 stability, and relieves CD276‐mediated T‐cell suppression. In vivo, WDR12 targeting sensitizes tumors to PD‐1 blockade, and combined SU14813 and anti‐PD‐1 therapy produces superior antitumor efficacy. These results define a WDR12‐CCT7‐CD276 axis that sustains immune resistance in melanoma and nominate WDR12 inhibition with PD‐1 blockade as a promising therapeutic strategy.

## Graphical abstract

WDR12 cooperates with the chaperonin subunit CCT7 to maintain CD276 stability on tumor cells, suppressing T‐cell activity and promoting immune escape. SU14813, a small‐molecule WDR12 inhibitor, reduces CD276 stability and relieves CD276‐mediated T‐cell suppression. Notably, WDR12 targeting sensitizes tumors to PD‐1 blockade, and combined SU14813 plus anti‐PD‐1 therapy produces superior antitumor efficacy.

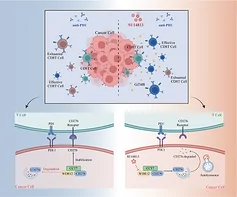

## 1 Introduction

Melanoma is a highly malignant tumor that originates from melanocytes and is one of the most lethal types of skin cancer. It exhibits a pronounced capacity for metastasis and is responsible for over 90% of skin cancer‐related deaths [1, 2, 3]. The emergence of immune checkpoint inhibitors (ICIs), such as anti‐PD‐1/PD‐L1 antibodies, has not only significantly prolonged the survival of patients with various tumors but also completely transformed the landscape of cancer treatment. However, for melanoma patients, the overall response rate of this therapy remains below 40%, and a proportion of initial responders develop drug resistance and eventually experience disease recurrence over time [4, 5, 6]. Tumors employ diverse mechanisms to remodel the immune microenvironment and evade immune surveillance [7, 8]. Therefore, identifying predictive biomarkers and elucidating the underlying mechanisms of drug resistance are critical for enhancing the efficacy of immunotherapy.

It has been observed that in patients with a poor response to conventional PD‐1/PD‐L1 inhibitors, there is an increased expression of alternative immune checkpoints (AICs), such as CD276 [9]. CD276 (also known as B7‐H3) belongs to the B7 immune regulatory protein family. It not only exerts immunosuppressive effects but also participates in the tumor progression process [10, 11]. It is overexpressed in various malignant tumors, where it inhibits CD8+ T‐cell‐mediated immune responses, thereby promoting tumor growth and metastasis. Blocking of CD276 enhances the antitumor function of CD8+ T cells and suppresses tumor development [12, 13, 14], making it a highly promising target for cancer immunotherapy [15, 16].

WD repeat domain 12 (WDR12) is a member of the functionally diverse WD‐repeat‐containing protein family in eukaryotic cells, featuring a structure of seven WD repeat domains [17]. WD proteins contain highly conserved repeating units that are flanked by characteristic N‐terminal GH (glycine‐histidine) and C‐terminal WD (tryptophan‐aspartic acid) dipeptides [18]. WDR12 is essential for processing the 32S precursor rRNA and facilitating the maturation of the 60S ribosomal subunit. Together with PES1 and BOP1, it forms a stable assembly known as the PeBoW complex—a key regulator of ribosome biogenesis in mammals [19]. Current studies have shown that WDR12 mainly regulates cell proliferation [20, 21, 22]. However, there is no substantive evidence to prove whether it can regulate the tumor immune microenvironment.

In this study, we found that WDR12 expression was upregulated in nonresponders to melanoma immunotherapy. After silencing WDR12, the stability of CD276 protein decreased, and it was degraded through the autophagy‐lysosome pathway, thereby significantly enhancing the cytotoxic function of CD8+ T cells. Moreover, we identified a small‐molecule compound targeting WDR12, named SU14813, which exhibits highly specific binding to WDR12 and enhances the activation state of CD8+ T cells by promoting the degradation of CD276. Importantly, the small molecule compound SU14813 not only synergistically enhances the efficacy of ICI but also counteracts immunotherapy resistance. Overall, our findings demonstrate that targeting WDR12 represents a highly promising novel therapeutic strategy for melanoma.

## 2 Results

### 2.1 Unraveling the Dynamic Evolution of Melanoma and the Predictive Value of WDR12 for Immunotherapy Response

**FIGURE 1:**
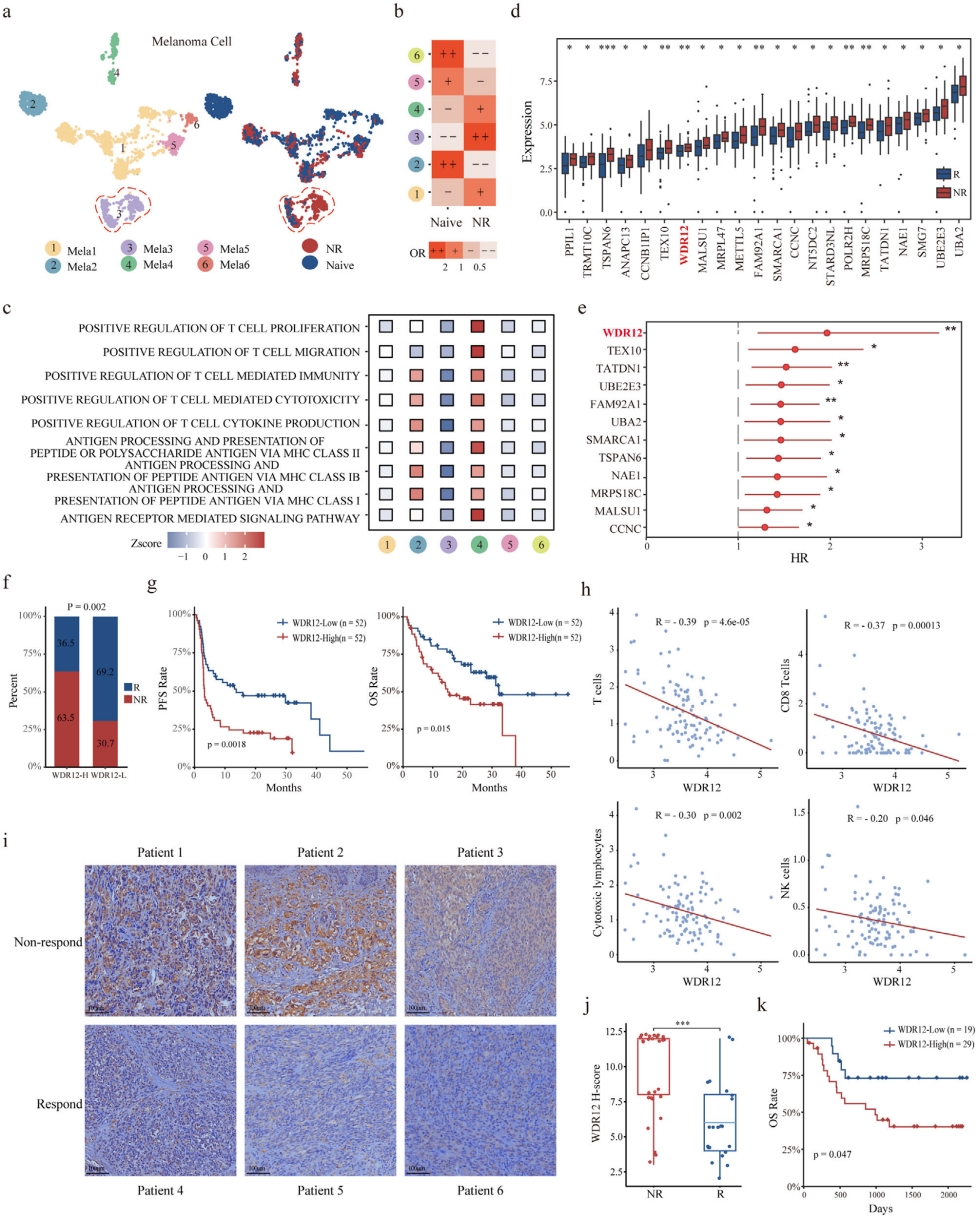
Unraveling the dynamic evolution of melanoma and the predictive value of WDR12 for immunotherapy response. (a) The UMAP visualization analysis shows the spatial distribution of six melanoma cell subpopulations (on the left), along with their corresponding treatment response profiles (on the right). (b) Analysis of the OR for tissue distribution propensity of cell subpopulation (OR > 2 indicates preferential distribution; OR < 0.5 indicates avoidance). (c) The differences in the activity of immune‐related pathways among cell subpopulations (the heatmap shows the Z‐score standardized expression). (d) The expression differences of the Mela3 characteristic genes in the anti‐PD‐1 treatment cohort (phs000452.v3.p1) between R and NR. (e) The forest plot presents the univariate Cox regression analysis of Mela3 characteristic genes based on PFS. f, The proportion of treatment response rate based on the expression level of WDR12 (WDR12‐H: high expression group, WDR12‐L: low expression group). (g) The Kaplan–Meier survival curve shows the impact of the WDR12 expression level on the PFS and OS of patients. (h) Correlation analysis of the WDR12 expression level and infiltration of antitumor immune cells. (i) Representative immunohistochemical images showing WDR12 expression in patients with different treatment responses (scale bar = 100 µm). (j) The Xiangya cohort verified the expression differences of WDR12 between R group (*n* = 21) and NR group (*n* = 27). (k) The Kaplan–Meier curve of OS for patients in the high/low expression group of WDR12. *p < 0.05, **p < 0.01, ***p < 0.001. NR, nonresponse to immunotherapy; R, response to immunotherapy; PFS, progression‐free survival; OS, overall survival.

The advent of immunotherapy has revolutionized the treatment landscape for melanoma. However, currently only a small proportion of melanoma patients respond to immunotherapy. To explore the possible reasons for the nonresponse to immunotherapy, using the single‐cell data published by Benjamin Izar [23], all cells were annotated as T cells, B cells, myeloid cells, fibroblasts, endothelial cells, and melanoma cells based on the expression levels of cell markers (Figure S1a–c). The focus of this study is on the tumor cells themselves. We divided all melanoma cells into six subgroups (Figure 1a). Among them, Mela3 accounted for as high as 30.7% in the nonresponse (NR) samples, while it was only 6.1% before treatment (Figure S1d). The odds ratio (OR) results also indicated that Mela3 was mainly enriched in the NR group (Figure 1b). Among the six subgroups, Mela3 showed the weakest activation state of lymphocytes and antigen‐presenting ability, resulting in the incomplete activation of effector T cells. As a result, its antitumor immune response was weakened (Figure 1c). Mela3 exhibits immunosuppressive effect, preventing it from being completely eliminated during PD‐1 inhibitor treatment. Its continuous proliferation results in a treatment nonresponse phenotype. Subsequently, we further explored the key molecules in Mela3. Among them, 22 molecules showed significantly higher expression in the NR group than in the response group (Figure 1d). Based on the results of the univariate Cox regression analysis, WDR12 demonstrated a significant correlation in both the progression‐free survival (PFS) and overall survival (OS). Its hazard ratio (HR) ranked first in the PFS, suggesting that WDR12 may play a crucial regulatory role in tumor immune escape (Figure 1e, Figure S1e,f). In the WDR12 low‐expression group (WDR12‐L), 69.2% of the samples showed a response to the PD‐1 inhibitor, while in the WDR12 high‐expression group (WDR12‐H), only 36.5% of the patients responded to the treatment (Figure 1f). Survival analysis indicated that the high expression of WDR12 was associated with poor prognosis—both in PFS and OS (Figure 1g). Furthermore, there were also significant differences in the infiltration of immune cells depending on the expression level of WDR12. The higher the expression level of WDR12, the lower the infiltration degree of various antitumor immune cells (Figure 1h). This might be the reason why only a few patients in the WDR12‐H group responded to immunotherapy. Consistent with this, immunohistochemical staining of melanoma samples from patients receiving PD‐1 inhibitor confirmed significantly higher WDR12 expression in nonresponders than in responders (Figure 1i,j). Through follow‐up, it was found that patients with a high expression level of WDR12 had a poorer prognosis (Figure 1k). Based on these results, we hypothesize that WDR12 plays an important role in the nonresponse to immunotherapy and can also serve as a predictor of the efficacy of immunotherapy.

### 2.2 Knockdown of WDR12 Promotes CD8+ T‐Cell Infiltration and Potentiates Antitumor Activity

**FIGURE 2:**
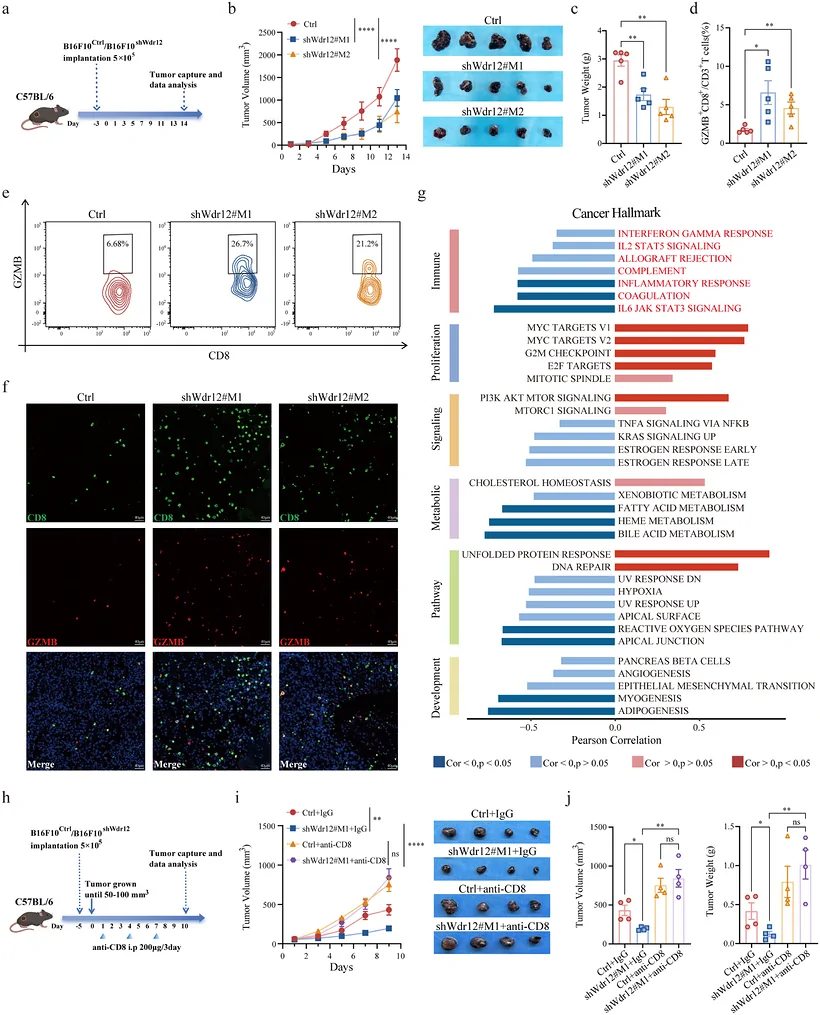
Knockdown of WDR12 promotes CD8+ T‐cell infiltration and potentiates antitumor activity. (a–g) C57BL/6 mice were subcutaneously inoculated with negative control (Ctrl) or shWdr12 (#M1 and #M2) treated B16F10 cells. (a) Schematic diagram of the mouse experimental design (*n* = 5). (b) Tumor growth curves. (c) Terminal tumor weight. (d) The proportion of GZMB+ CD8+ T cells in CD3+ TILs. (e) Representative flow cytometry gating strategy showing GZMB expression in CD8+ T cells. (f) Representative images of CD8 (Green) and GZMB (Red) IF staining in tumor tissues (the cell nuclei stained with DAPI, scale bar = 40 µm). (g) The correlation between the WDR12 expression levels and cancer hallmarks scores was assessed using proteomic data from mice. (h–j) The shWdr12‐B16F10 tumor‐bearing mice were treated with anti‐CD8 antibody or isotype IgG. (h) Schematic diagram of the mouse experimental design (*n* = 4). (i) Tumor growth curves. (j) Terminal tumor volume (left) and terminal tumor weight (right). All data are presented as mean ± SEM. The *p*‐values were calculated using the unpaired, two‐tailed *t*‐test and two‐way ANOVA analysis. Not significant (ns), *p* > 0.05; *p < 0.05; **p < 0.01; ***p < 0.001. TILs, tumor‐infiltrating lymphocytes; GZMB, granzyme B.

Previous studies have reported that WDR12 acts as a tumor‐promoting factor by regulating tumor‐cell proliferation [20, 21, 24], but its immunomodulatory role in the tumor microenvironment remains unclear. To explore the immunomodulatory effect of WDR12, we established a stable WDR12 knockdown B16F10 melanoma cell line. WDR12 knockdown had a minimal effect on tumor growth, as demonstrated by both CCK‐8 assays and in vivo experiments using NSG mice bearing WDR12‐knockdown B16F10 tumors (Figure S2a–d). However, in immunocompetent C57BL/6 mice, WDR12 knockdown significantly suppressed tumor growth (Figure 2a–c), suggesting that WDR12 may play a critical role in the tumor microenvironment. Therefore, we further assessed the infiltration of T cells in melanoma. We found that downregulation of WDR12 significantly increased both the number of intratumoral CD8+ T cells and their Granzyme B‐mediated killing activity (Figure 2d,e; Figure S2e,f). To better visualize the infiltration of cytotoxic CD8+ T cells into the tumor, we performed immunofluorescence (IF) staining of tumor sections. Our results confirmed that the absence of WDR12 did indeed lead to an increase in tumor‐infiltrating cytotoxic CD8+ T cells (Figure 2f). At the same time, we conducted proteomic sequencing on the tumor tissues of mice. The data revealed that WDR12 expression was negatively correlated with multiple tumor immune‐related pathways (Figure 2g). To further confirm that WDR12 regulates tumor immunity through CD8+ T cells, we reinjected WDR12 knockdown B16F10 melanoma cells into C57BL/6 mice, and used anti‐CD8α antibodies (mAb) to deplete CD8+ T cells. Notably, after treatment with the anti‐CD8α antibodies, the tumor burden difference caused by WDR12 knockdown was abrogated (Figure 2h–j), and there was no significant fluctuation in the weight of the mice (Figure S2g). Additionally, flow cytometry confirmed that CD8+ T cells in the mouse tumors were depleted (Figure S2h). Taken together, these findings indicate that WDR12 exerts tumor immune regulatory functions by depending on CD8+ T cells.

### 2.3 Knockdown of WDR12 Reduces CD276, the Level of Which Correlates With the Efficacy of Immunotherapy

**FIGURE 3:**
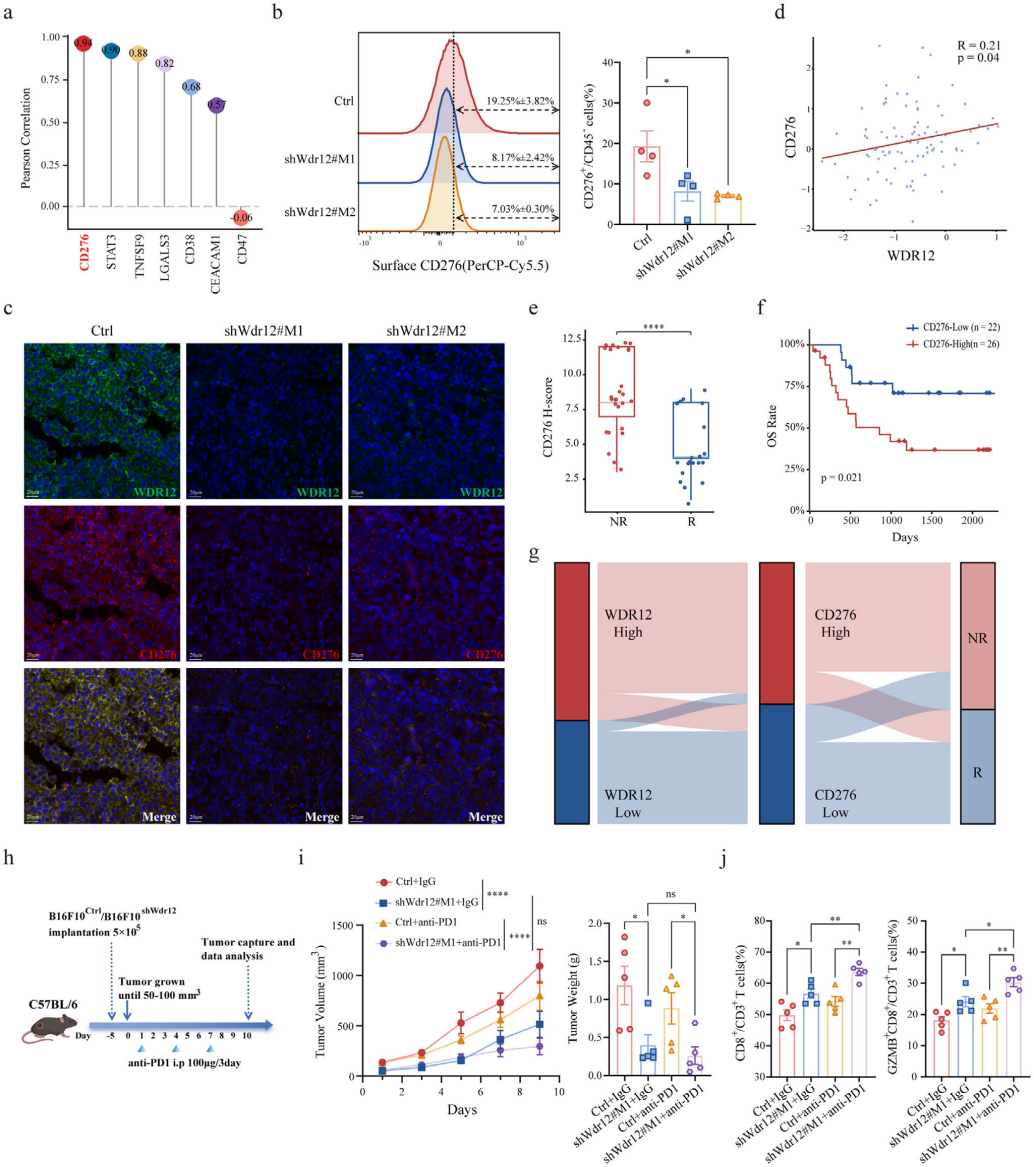
Knockdown of WDR12 reduces CD276, the level of which correlates with the efficacy of immunotherapy. (a) The correlation between WDR12 expression and immune regulatory molecules was analyzed using mouse proteomic data. (b) The expression of CD276 on the cell membrane of CD45−‐ cells in tumor tissues was detected by flow cytometry. (c) Representative images of WDR12 (Green) and CD276 (Red) IF staining in tumor tissues (The cell nuclei stained with DAPI, scale bar = 20 µm). (d) The correlation between the expression of WDR12 and CD276 in the proteomics of patients with melanoma immunotherapy. (e) The Xiangya cohort verified the expression differences of CD276 between the R group (*n* = 21) and the NR group (*n* = 27). (f) The Kaplan–Meier curve of OS for patients in the high/low expression group of CD276. (g) Sankey diagram demonstrated the association between the WDR12‐CD276 co‐expression network and the response to immunotherapy. (h–j) The shWdr12‐B16F10 tumor‐bearing mice were treated with anti‐PD‐1 antibody or isotype IgG. (h) Schematic diagram of the mouse experimental design (*n* = 5), (i) tumor growth curves (left) and terminal tumor weight (right). (j) The proportion of CD8+ and GZMB+ CD8+ T cells in CD3 TILs. All data are presented as mean ± SEM. The *p*‐values were calculated using the unpaired, two‐tailed *t*‐test and two‐way ANOVA analysis. Not significant (ns), *p* > 0.05; *p < 0.05; **p < 0.01; ***p < 0.001.

Extensive evidence demonstrates that tumor cells can suppress the antitumor activity of CD8+ T cells by upregulating inhibitory immune checkpoint molecules [6]. To deeply explore how WDR12 affects the activity of T cells. Firstly, through the proteomics analysis of mice, we found that the expression of WDR12 was significantly positively correlated with multiple immune regulatory molecules. Among them, CD276 had the strongest correlation (Pearson *r* = 0.94) (Figure 3a; Figure S3a). To verify this finding, we used flow cytometry to detect the expression of several immune checkpoint molecules on the surface of tumor cells. The results showed that after WDR12 knockdown, the expression level of the CD276 decreased the most significantly (Figure 3b; Figure S3b). IF staining provided additional confirmation of this result. In WDR12‐knockdown tumor tissues, the fluorescence intensity of CD276 was weaker than in the control group (Figure 3c). To confirm the clinical correlation between WDR12 and CD276, we analyzed proteomic data from a melanoma immunotherapy cohort [25], which revealed a positive correlation between them (Figure 3d). Furthermore, immunohistochemical analysis of clinical samples showed that CD276 expression was significantly higher in nonresponders than in responders, and high CD276 expression was associated with shorter OS (Figure 3e,f; Figure S3c). Consistently, we observed a significant positive correlation between WDR12 and CD276 expression (*r* = 0.8), which was further supported by a Sankey diagram indicating their co‐expression pattern (Figure 3g; Figure S3d). These results suggest that WDR12 positively regulate the expression of CD276, thereby facilitating tumor immune escape and playing a vital role in the mechanism of ineffective immunotherapy. To assess the potential role of WDR12 in immunotherapy, we administered anti‐PD‐1 antibody or an equal amount of IgG to C57BL/6 mice that had been injected with WDR12‐knockdown B16F10 (shWdr12‐B16F10) cells. Compared with the anti‐PD‐1 monotherapy group, the anti‐PD‐1‐treated shWdr12 group exhibited significant tumor growth inhibition without affecting mouse body weight, demonstrating that WDR12 deficiency enhances the antitumor efficacy of anti‐PD‐1 therapy. Within the shWdr12 group, although anti‐PD‐1 treatment showed only a trend toward growth inhibition relative to the IgG control group (*p* > 0.05), it led to a marked increase in intratumoral cytotoxic CD8+ T‐cell infiltration, suggesting that anti‐PD‐1 therapy remains capable of effectively activating immune responses in the context of WDR12 deficiency (Figure 3h–j; Figure S3e,f). Together, these data indicate that the combination of WDR12 targeting and PD‐1 blockade may represent a promising antitumor therapeutic strategy.

### 2.4 WDR12 Knockdown Degrades CD276 Via the Autophagy‐Lysosome Pathway

**FIGURE 4:**
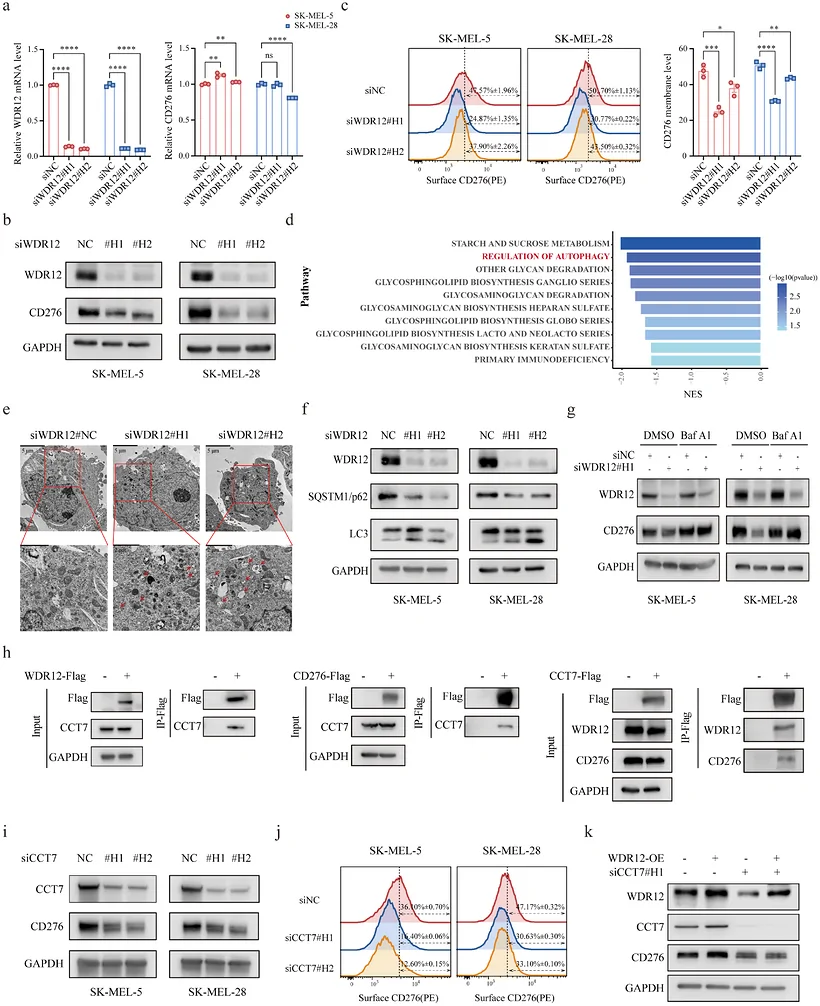
WDR12 requires the molecular chaperone CCT7 to stabilize the expression of CD276. (a–c) Analysis of CD276 expression in melanoma cell lines (SK‐MEL‐5 and SK‐MEL‐28) transfected with siNC or siWDR12 (#H1 and #H2). (a) RT‐qPCR, (b) Western blotting analysis of the expression of WDR12 or CD276, and (c) FACS analysis of CD276 expression levels. (d) Functional enrichment analysis of down‐regulated proteins based on mouse proteomics data. (e) Representative TEM images of SK‐MEL‐28 cells transfected with siNC or siWDR12 (red arrows: autolysosome). (f) Western blotting analysis of SQSTM1/p62 and LC3 expression in melanoma cell lines transfected with siNC or siWDR12. (g) Western blotting analysis of CD276 expression in melanoma cells treated with Baf A1 (400 nM) for 4 h. (h) Co‐IP confirmed the interaction among WDR12, CD276, and CCT7. (i) Western blotting analysis of CD276 expression in melanoma cell lines transfected with siNC or siCCT7 (#H1 and #H2). (j) FACS analysis of CD276 expression in melanoma cell lines transfected with siNC or siCCT7. (k) Western blotting analysis of CD276 expression upon WDR12 overexpression and CCT7 knockdown. All data are presented as mean ± SEM and were obtained from three independent experiments. The *p*‐values were calculated using the unpaired, two‐tailed *t*‐test. Not significant (ns), *p* > 0.05; *p < 0.05; **p < 0.01; ***p < 0.001. Baf A1, Bafilomycin A1.

To elucidate the molecular mechanism by which WDR12 regulates CD276 expression, we knocked down WDR12 using small interfering RNA (siRNA) in the human melanoma cell lines SK‐MEL‐5 and SK‐MEL‐28. WDR12 knockdown led to a marked decrease in both total and membrane CD276 protein levels, whereas no concomitant alteration was detected at the mRNA level (Figure 4a–c; Figure S4a). In contrast, WDR12 overexpression significantly increased the level of CD276 protein (Figure S4b). Hence, it can be inferred that WDR12 regulates CD276 expression at the translational or posttranslational level. Based on the known function of WDR12 in ribosome biogenesis [19, 26, 27], we subsequently investigated whether it regulates the expression of CD276 through this pathway. By specifically knocking down other components of the PeBoW complex, PES1 or BOP1, or by treating with the RNA polymerase I inhibitor CX‐5461 [22], no changes in the expression level of CD276 protein were observed (Figure S4c,d). These results imply that WDR12 may have a new function independent of ribosome biogenesis. Of note, proteomic data showed that proteins downregulated by WDR12 knockdown were significantly enriched in the autophagy pathway, indicating a potential role for WDR12 in autophagy regulation (Figure 4d). Consistently, transmission electron microscopy (TEM) revealed an increased number of autolysosomes in WDR12‐deficient cells (Figure 4e). At the molecular level, detection of autophagy markers showed that knockdown of WDR12 led to a decrease in SQSTM1/p62 protein levels, while LC3‐II levels increased (Figure 4f; Figure S4e). These results collectively confirm that WDR12 deficiency promotes autophagy in melanoma. To determine whether WDR12 regulates the degradation of CD276 via the autophagy‐lysosome pathway, we treated WDR12‐knockdown cells with the autophagy inhibitor Bafilomycin A1 (Baf A1) and found that it could restored CD276 protein levels that had been decreased by WDR12 knockdown (Figure 4g). These findings confirmed that WDR12 downregulation promotes the degradation of CD276 via the autophagy‐lysosome pathway.

### 2.5 WDR12 Requires CCT7 to Stabilize CD276 Protein Expression

To illuminate the molecular mechanism underlying the stable expression of CD276 by WDR12, we generated melanoma cell lines stably expressing WDR12‐Flag or CD276‐Flag using lentiviral transduction. Immunoprecipitation (IP) with Flag magnetic agarose followed by mass spectrometry (MS) identified a total of 11 proteins (excluding ribosomal‐related proteins) interacting with WDR12 and CD276 (Figure S4f). It is notable that three of the identified proteins (CCT7, CCT8, and Rab5C) are linked to the autophagy pathway [28, 29, 30]. Subsequent co‐immunoprecipitation (Co‐IP) assays validated that only CCT7 could directly binds to both WDR12 and CD276 (Figure 4h; Figure S4g), suggesting that CCT7 serves as an essential bridging adaptor between WDR12 and CD276. CCT7, as one of the core subunits of the eukaryotic chaperonin TCP‐1 ring complex (TRiC/CCT), is involved in the proper folding of approximately 10%–15% of cytosolic proteins [31, 32, 33]. Furthermore, CCT7 knockdown was found to reduce the total and membrane protein expression levels of CD276, while no corresponding change was observed at the transcriptional level (Figure 4i,j; Figure S4h,i). This provides evidence that CCT7 has the ability to stabilize CD276. To assess whether WDR12 requires CCT7 to maintain CD276 stability, we knocked down CCT7 in WDR12‐overexpressing cells. The results showed that CD276 expression decreased regardless of WDR12 status (Figure 4k). In summary, our experimental data confirm that CCT7 is essential for WDR12 to maintain CD276 stability.

### 2.6 The Small‐Molecule Compound SU14813 Targeting WDR12 Downregulates CD276 and Potentiates CD8+ T‐Cell Antitumor Function

**FIGURE 5:**
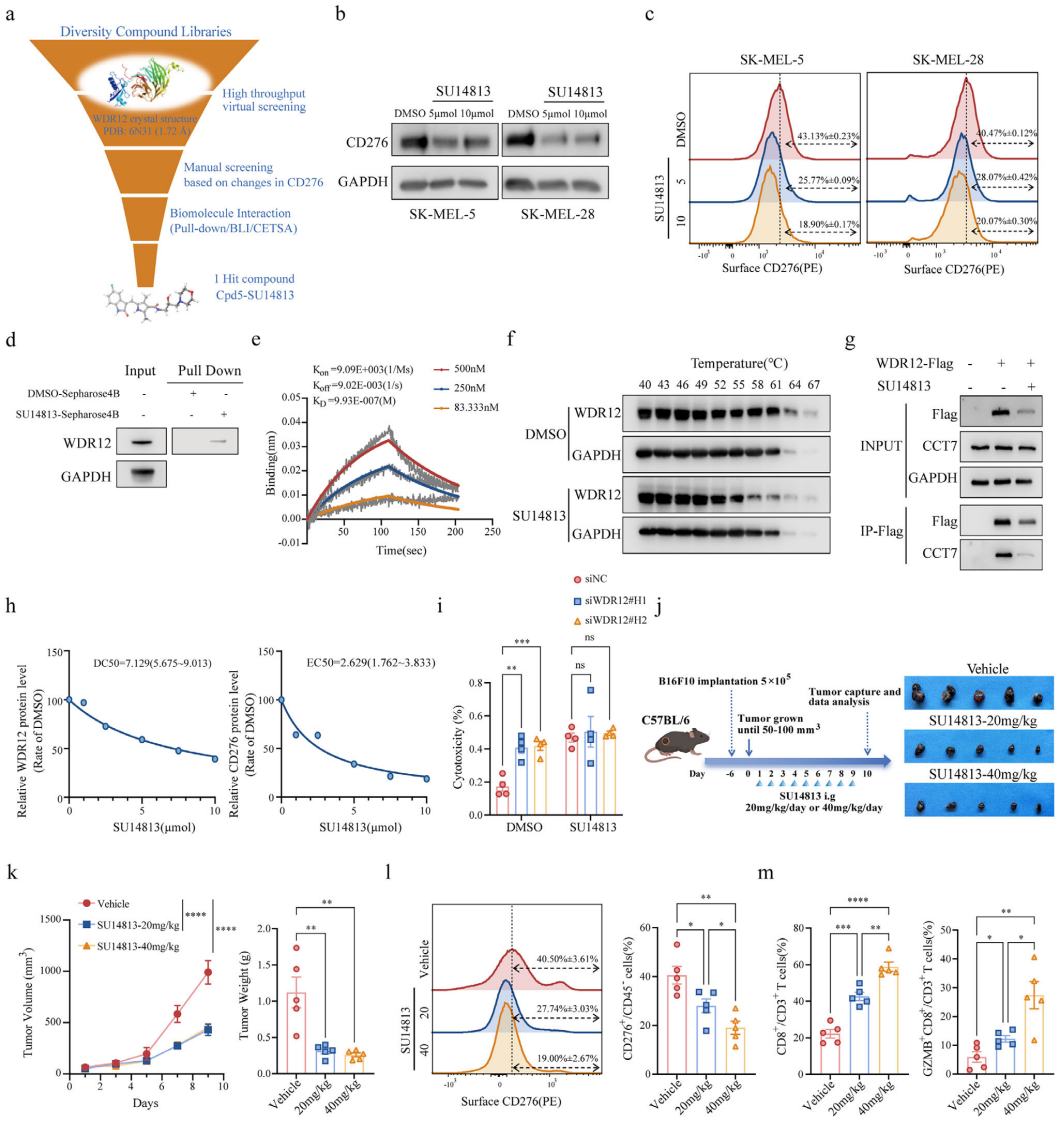
The small molecule compound SU14813 targeting WDR12 down‐regulates CD276 and potentiates CD8+ T‐cell antitumor function. (a) Schematic diagram of the computer‐assisted drug screening. (b) Western blotting analysis of CD276 expression in melanoma cell lines treated with SU14813 for 48 h. (c) FACS analysis of CD276 expression in melanoma cell lines treated with SU14813 for 48 h. (d) Pull‐down analysis of SU14813 and WDR12 interaction. (e) BLI monitors the interaction between SU14813 and WDR12. (f) CETSA analysis of SU14813 on the thermal stability of target protein WDR12. SK‐MEL‐28 cells were treated with DMSO or SU14813 (10 µM) for 48 h and then heated at the indicated temperatures. (g) Co‐IP assay analyzed the interaction between WDR12 and CCT7 after treatment with SU14813. (h) Determine the DC50 and EC50 of SU14813 on WDR12. (i) T cell‐mediated tumor cell‐killing assay. SK‐MEL‐28 cells were cocultured with activated T cells (1:2) for 24 h, followed by luciferase assay. (j–m) The B16F10 tumor‐bearing mice were treated with SU14813 or vehicle. (j) The experimental design for SU14813 treatment (*n* = 5), (k) tumor growth curve (left) and terminal tumor weight (right). (l) The expression of CD276 on the cell membrane of CD45− cells in tumor tissues was detected by flow cytometry. (m)The proportion of CD8+ and GZMB+ CD8+ T cells in CD3 TILs. All data are presented as mean ± SEM. The *p*‐values were calculated using the unpaired, two‐tailed *t*‐test and two‐way ANOVA analysis. Not significant (ns), *p* > 0.05; *p < 0.05; **p < 0.01; ***p < 0.001. BLI, bio‐layer interferometry; CETSA, cellular thermal shift assay; EC50, half effective concentration; DC50, half degradation concentration.

Our data suggest that WDR12 could regulate the degradation of CD276, positioning WDR12 as a potential therapeutic target for melanoma. Therefore, the small‐molecule compound targeting WDR12 could not only offer a novel treatment approach but also demonstrate synergistic efficacy when combined with the PD‐1 inhibitor. Of the top 30 candidate compounds identified through computer‐aided drug screening, SU14813(Cpd5) exhibited the greatest potency in downregulating CD276 in vitro (Figure 5a–c; Figure S5a and b). The results of molecular docking and molecular dynamics simulations revealed that SU14813 could bind to the active site of WDR12. Specifically, it formed hydrophobic interactions with residues ILE195, ALA196, VAL197, VAL263, and LEU264. Hydrogen bonds were formed with SER105, SER391, VAL263, and TRP265, and a π‐cation interaction was observed with LYS108 (Figure S5c). Next, to verify the interaction between SU14813 and WDR12, we incubated melanoma cell lysates with an SU14813‐Sepharose 4B agarose complex. The pull‐down experiment showed that WDR12 could bind to the SU14813‐Sepharose 4B complex, whereas no binding signal was detected in DMSO‐Sepharose 4B control (Figure 5d). Next, Bio‐layer interferometry (BLI) was used to quantitatively analyze the interaction kinetics. The measured equilibrium dissociation constant (KD) was 993 nM, with a dissociation rate constant (Koff) of 9.02 × 10^−3^ s^−1^, indicating that the SU14813‐WDR12 complex is relatively stable and exhibits high affinity (Figure 5e). To validate the binding of SU14813 to endogenous WDR12 under physiologically conditions, we performed a cellular thermal shift assay (CETSA), which monitors compound‐induced changes in protein thermal stability. Melanoma cells were treated with DMSO or SU14813 for 48 h and then subjected to a temperature gradient ranging from 40°C to 67°C. As shown in Figure 5f and Figure S5d, SU14813 binding significantly reduced the thermal stability of WDR12, decreasing the Tm from 63.63°C in the control group to 57.38°C in the SU14813‐treated group. This negative ΔTm is consistent with the thermal destabilization induced by targeted protein degraders (e.g., Molecular glues, PROTACs), suggesting that SU14813 may promote WDR12 degradation. Further experiment revealed that SU14813 downregulated the WDR12 protein levels in a concentration‐dependent manner and impaired its binding to CCT7 (Figure 5g,h; Figure S5e). Overall, these results indicate that SU14813 binding to WDR12 could facilitate its degradation (DC50 = 7.129 µM) by inducing conformational changes, thereby suppressing its biological function (EC50 = 2.629 µM). To determine the degradation pathway, we treated melanoma cells with the MG132 (proteasome inhibitor) or Baf A1. Our result showed that SU14813‐induced WDR12 degradation was effectively blocked by MG132 but not by Baf A1, indicating that SU14813 promotes WDR12 degradation predominantly through the ubiquitin‐proteasome pathway (Figure S5f). Taken together, these results indicate that SU14813 possesses molecular glue properties, enabling it to promote targeted degradation of WDR12 and subsequently downregulate CD276 expression. To evaluate the antitumor activity of SU14813 in vitro and in vivo, we first performed a T‐cell‐mediated cytotoxicity assay. Knockdown of WDR12 significantly enhanced T‐cell‐mediated antitumor activity, while SU14813 treatment abolished the difference in cytotoxicity between the control and knockdown groups. These results indicate that the antitumor activity of SU14813 is mediated by WDR12 (Figure 5i). Subsequently, we established a C57BL/6 mouse model via subcutaneous inoculation of wild‐type B16F10 cells (B16F10‐WT) to assess the in vivo efficacy of SU14813. We found that compared with the vehicle group, SU14813 treatment significantly inhibited tumor growth and prolonged mouse survival (Figure 5j,k; Figure S5g–i). Body weight remained stable without significant changes throughout the treatment period. Furthermore, no significant abnormalities were observed between the vehicle and SU14813‐treated groups in plasma biochemical parameters (ALT, AST, CRE, BUN, LDH, and CK) or in the histopathological examination of major organs (heart, liver, lung, spleen, and kidney), indicating a favorable safety profile for SU14813 under the evaluated conditions (Figure S5j–l). Consistent with in vitro findings, flow cytometry showed that CD276 expression decreased dose‐dependently in response to increasing concentrations of SU14813 (Figure 5l). Notably, the proportion of intratumoral cytotoxic CD8+ T cells rose in a parallel dose‐dependent manner (Figure 5m). Together, these data demonstrate that SU14813 exerts its antitumor activity by targeting the WDR12‐CCT7‐CD276 axis to modulate the tumor immune microenvironment.

### 2.7 The Dual Role of SU14813: Boosting Anti‐PD‐1 Antibody Efficacy While Overcoming Resistance

**FIGURE 6:**
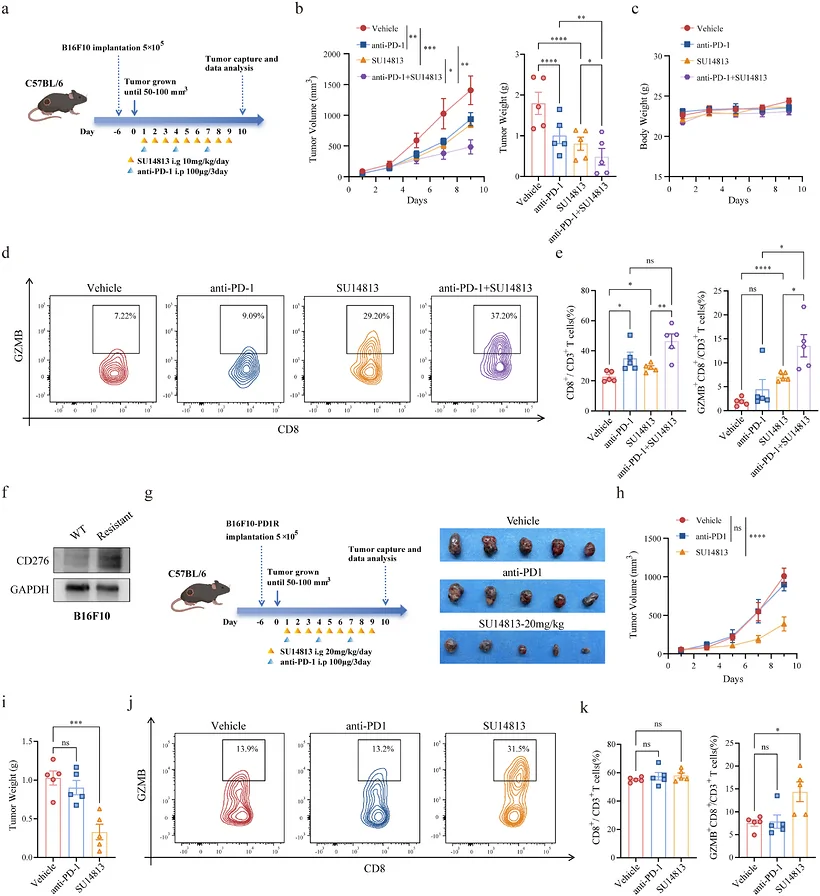
The dual role of SU14813: Boosting anti‐PD‐1 antibody efficacy while overcoming resistance. (a–e) The B16F10 tumor‐bearing mice were treated with anti‐PD‐1 antibody, SU14813, or combination. (a) Schematic diagram of the experimental design (*n* = 5), (b) tumor growth curves (left) and terminal tumor weight (right). (c) Monitoring of the mice's body weight during the treatment period. (d) Representative flow cytometry gating strategy showing GZMB expression in CD8+ T cells. (e) The proportion of CD8+ and GZMB+ CD8+ T cells in CD3 TILs. (f) Western blotting analysis of CD276 expression in B16F10‐WT cells and B16F10‐PD1R cells. (g–k) The B16F10‐PD1R tumor‐bearing mice were treated with anti‐PD‐1 antibody, SU14813 or vehicle. (g) Schematic diagram of the experimental design (*n* = 5), (h) tumor growth curves, and (i) terminal tumor weight. (j) Representative flow cytometry gating strategy showing GZMB expression in CD8+ T cells. (k) The proportion of CD8+ and GZMB+ CD8+ T cells in CD3 TILs. All data are presented as mean ± SEM. The *p*‐values were calculated using the unpaired, two‐tailed *t*‐test and two‐way ANOVA analysis. Not significant (ns), *p* > 0.05; *p < 0.05; **p < 0.01; ***p < 0.001.

To evaluate the antitumor efficacy of the combination of SU14813 and anti‐PD‐1 antibody, we subcutaneously inoculated B16F10‐WT cells into C57BL/6 mice and then treated with anti‐PD‐1 antibody, SU14813, or the combination (Figure 6a). Compared with monotherapy, combination treatment with SU14813 and anti‐PD‐1 antibody exhibited superior antitumor efficacy without causing any changes in mouse body weight (Figure 6b,c). Flow cytometry further revealed that the combination treatment led to greater infiltration of cytotoxic CD8+ T cells than monotherapy (Figure 6d,e). Moreover, we sought to elucidate the potential role of SU14813 in immunetherapy resistance. First, we established an anti‐PD‐1 antibody resistant B16F10 cell line (B16F10‐PD1R) through multiple rounds of in vivo selection. Notably, we observed that the expression level of CD276 in B16F10‐PD1R cells was higher than that in the parental B16F10‐WT cells (Figure 6f). Next, we used B16F10‐PD1R cells to establish a subcutaneous tumor‐bearing model in C57BL/6 mice, and then treated the mice with anti‐PD‐1 antibody or SU14813. SU14813 significantly reduced tumor volume and weight, whereas anti‐PD‐1 antibody treatment failed to show any significant effect compared with vehicle (Figure 6g–i). This not only validated the reliability of the anti‐PD‐1 resistance model, but also confirmed that SU14813 could effectively overcome the resistance to anti‐PD‐1 antibody. Mechanistically, SU14813 treatment did not affect CD8+ T‐cell infiltration but significantly elevated Granzyme B expression. (Figure 6j,k). These data clearly demonstrate that SU14813 possesses dual capabilities: synergizing with anti‐PD‐1 antibody to enhance antitumor efficacy and combating its resistance.

## 3 Discussion

**FIGURE 7:**
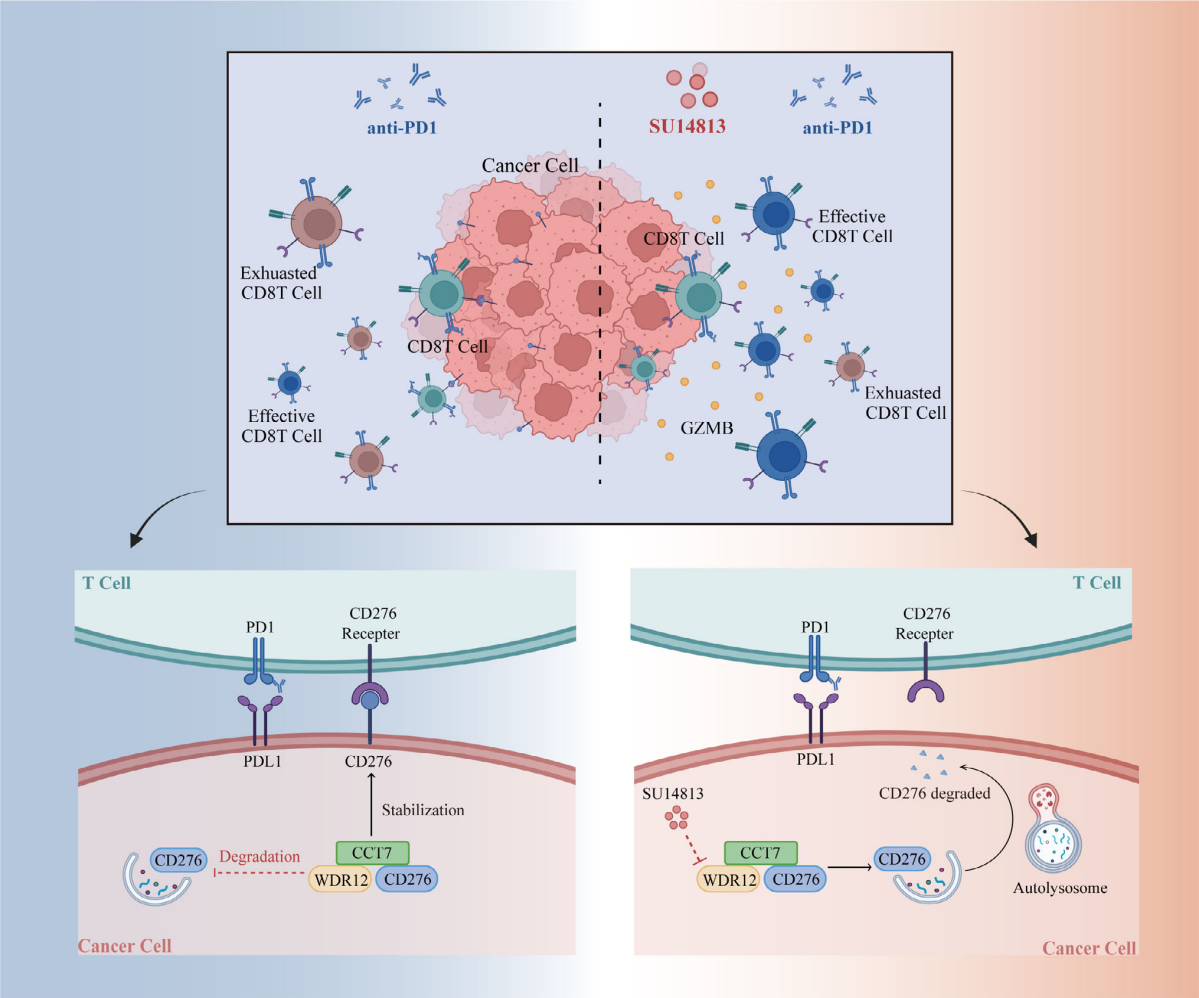
A schematic diagram illustrating the mechanism by which WDR12, through the assistance of molecular chaperone CCT7, stabilizes the expression of CD276 and promotes immune escape in melanoma. The graph was drawn with biorender (https://www.biorender.com/).

This study for the first time clearly demonstrates a significant correlation between high expression of WDR12 and nonresponse to PD‐1 inhibitor in melanoma. Mechanistically, WDR12 requires the molecular chaperone CCT7 to bind the immune checkpoint protein CD276, forming a triple complex. This complex inhibits the degradation of CD276 through the autophagy‐lysosome pathway, enabling it to be stably expressed on the surface of tumor cells and thereby continuously exerting an immunosuppressive effect. After targeting WDR12, the complex failed to form, resulting in the degradation of CD276 and enhancing the antitumor efficacy of CD8+ T cells (Figure 7). In this study, we revealed a new function of WDR12 in regulating CD276 protein stability, which is independent of the classical pathway. This discovery provides a new perspective for understanding of the biological function of WDR12.

Immune checkpoints play a crucial role in maintaining immune homeostasis, preventing excessive activation of T cells and the consequent immune‐mediated damage to the host [34, 35]. Tumor cells can exploit this mechanism by expressing inhibitory checkpoint molecules to suppress the functions of immune cells, thereby evading immune surveillance. Clinically, PD‐1/PD‐L1 inhibitors have significantly improved the survival rates of patients with various tumors. However, a large number of patients remain nonresponsive to these therapies, suggesting that the mechanism of immune escape extends beyond the PD‐1/PD‐L1 pathway [36, 37]. By integrating single‐cell and bulk RNA‐seq data from PD‐1 inhibitor, we identified WDR12 as a key target strongly associated with nonresponse to immunotherapy, suggesting that WDR12 may serve as a novel predictive biomarker for the response to immunotherapy. Previous studies have reported that WDR12 plays a tumor‐promoting role in various malignancies [20, 21]; however, its function in the tumor immune microenvironment has not been systematically elucidated. In this study, we reveal for the first time a novel immune escape mechanism involving WDR12, providing new insights into its role in tumor immune regulation.

Proteomic analysis showed that there was a strong positive correlation between the protein levels of WDR12 and CD276 in tumor tissues, suggesting that WDR12 might inhibit the activation of CD8+ T cells by stabilizing CD276, thereby promoting tumor progression. CD276 plays distinct immunomodulatory roles in autoimmune disease and cancer. It exerts a pro‐inflammatory effect in autoimmune disease models, whereas it plays an inhibitory role in the tumor microenvironment [16, 38]. In this study, we further identified that CCT7 is a key molecular determinant in the regulation process of CD276 by WDR12. CCT7, as the core subunit of the eukaryotic molecular chaperone complex TRiC/CCT, plays an important role in protein folding and the maintenance of protein homeostasis [39, 40]. WDR12 is capable of maintaining the stability of CD276, and this process requires the assistance of CCT7.

Compelling evidence supports targeting CD276 as a novel and promising antitumor therapeutic strategy. Currently, most existing clinical trials targeting CD276 employ antibody‐based therapies [38]. However, these therapies have certain limitations, such as poor penetration of tumor tissues, high costs, and potential immune‐related adverse effects. In contrast, small molecule drugs offer distinct advantages, including high levels of tissue penetration, oral administration, and more predictable and easily monitored drug metabolism [41, 44]. Therefore, the development of specific small‐molecule inhibitors represents a highly promising and effective alternative therapeutic approach. Unfortunately, current understanding of the CD276 signaling pathway, especially its potential unknown receptors or functional binding partners, remains markedly inadequate [45]. This critical bottleneck severely restricts the progress of developing small molecule drugs targeting CD276.

This study has elucidated the molecular mechanism by which WDR12 stabilizes CD276 expression, indicating that WDR12 is a promising target. Therefore, the development of small molecule compound targeting WDR12 is expected to achieve precise regulation of CD276. Based on this, we utilized molecular docking and drug screening to identify the small molecule compound SU14813, which specifically binds to WDR12. This binding prevents the assembly of the WDR12‐CCT7‐CD276 triple complex, reduces the expression of CD276 on tumor cells, and potently enhances the antitumor effects of PD‐1 blocking therapy. SU14813 is an oral multi‐target tyrosine kinase inhibitor (TKI) that specifically inhibits vascular endothelial growth factor receptors (VEGFRs), platelet‐derived growth factor receptors (PDGFR), stem cell factor receptors (KIT), and FMS‐like tyrosine kinase 3 (FLT3). In terms of structure and target sites, this drug is similar to sunitinib; however, SU14813 possesses some unique advantages, such as a shorter plasma half‐life, no detectable pharmacologically active metabolites in circulation, 40% oral bioavailability, and linear pharmacokinetic characteristics. These properties collectively contribute to its favorable safety and controllable tolerability [46, 47]. Importantly, distinct from conventional kinase inhibitors, SU14813 induces the degradation of its target protein WDR12 via the ubiquitin‐proteasome pathway, thereby reducing the protein level of CD276. This study demonstrates for the first time that SU14813 possesses molecular glue degrader activity, establishing a new paradigm for ubiquitination‐based degradation strategies targeting CD276. Together, these findings reveal a noncanonical mechanism of SU14813 and provide an important theoretical foundation for future drug optimization.

Some clinical patients showed a partial response (PR) to the PD‐1 inhibitor during early stage but still experienced tumor recurrence with prolonged therapy [48]. Based on this phenomenon, we applied SU14813 to a mouse model resistant to PD‐1 blockade therapy. The results showed that SU14813 significantly inhibited tumor growth. These findings indicate that SU14813 not only has the potential to serve as an independent treatment for melanoma, but also shows great prospects for combination with existing ICI, making it a highly valuable candidate drug.

## 4 Materials and Methods

### 4.1 Ethical Statement

All human and animal studies were conducted in compliance with relevant ethical guidelines and were approved by the appropriate ethics committees. The research protocol (Protocol Number: 2024070866) was approved by the Medical Ethics Committee of Xiangya Hospital, Central South University. All human tissue samples were obtained in accordance with informed consent policies, and written informed consent was provided by all participants. The study adhered to the principles set forth in the Declaration of Helsinki. All animal experiments were approved by the Institutional Animal Care and Use Committee (IACUC) of Central South University (Protocol Number: CSU‐2023‐0130) and were carried out in compliance with institutional guidelines.

### 4.2 OR

To characterize the distribution patterns of melanocyte subpopulations across different tissues, we adopted the OR method described by Zhang et al. [49] to assess tissue distribution preferences. An OR value greater than 2 indicates a preferential distribution of the subpopulation in the corresponding tissue, while an OR value less than 0.5 suggests no distribution preference for that tissue.

### 4.3 Analysis of Functional and Immune Characteristics

The MCP counter was employed to evaluate the immune cell infiltration levels in the ICI bulk transcriptome data. The proteomics data generated in this study from mouse models were normalized, log‐transformed, and subsequently subjected to Gene Set Variation Analysis (GSVA) to calculate the activity levels of Hallmark pathways in each sample. Gene sets associated with positive regulation of T‐cell activation and antigen presentation functions were collated to assess the functional characteristics of six melanoma subgroups. The gene sets “h.all.v2023.2.Hs.symbols” and “c2.cp.kegg.v7.5.1.symbols” were retrieved from the MSigDB database (http://software.broadinstitute.org/gsea/msigdb/index.jsp). Enrichment analysis was performed using the “clusterProfiler” R package.

### 4.4 Clinical Data and Sample Collection

This study collected paraffin‐embedded tumor tissue sections and corresponding clinical data from 48 melanoma patients who received PD‐1 inhibitors at Xiangya Hospital, Central South University, between March 2019 and March 2024. The research protocol was approved by the Medical Ethics Committee of Xiangya Hospital, Central South University. All tissue samples were obtained with written informed consent from the patients, and all participants were followed for at least 6 months. According to the RECIST 1.1 criteria, patients who achieved a complete response (CR), PR, or stable disease (SD) with PFS longer than 6 months were classified as the response group (Rs). Patients with SD but a PFS of ≤6 months, or with progressive disease (PD), were classified as the NRs.

### 4.5 Cell Culture

All cell lines were obtained from the American Type Culture Collection (ATCC). The human malignant melanoma cell lines SK‐MEL‐5 and SK‐MEL‐28, as well as the HEK293T cell line, were cultured in Dulbecco's modified Eagle's medium (Gibco) supplemented with 10% fetal bovine serum (ExCell Bio) and 1% penicillin–streptomycin solution (Beyotime Biotechnology). Cells were maintained at 37°C in a humidified incubator with 5% CO_2_. The mouse malignant melanoma cell line B16F10 was cultured in the RPMI‐1640 medium (Gibco) under the same conditions.

### 4.6 Antibodies and Chemicals

Anti‐WDR12(ab95070) and anti‐CD276(ab315907) were from Abcam. anti‐CD276(14058T), anti‐SQSTM1/P62(8025T), anti‐LC3A/B(12741T), and anti‐DYKDDDDK Tag(14793T) were from Cell Signaling Technology. anti‐CCT7(67540‐1‐AP), anti‐PES1(13553‐1‐AP), anti‐BOP1(28366‐1‐AP), and anti‐GAPDH(60004‐1‐Ig) were from Proteintech. Anti‐WDR12(HPA036389) was from Sigma‐Aldrich. Anti‐mouse PD‐1‐InVivo (A2122) and anti‐mouse CD8a‐InVivo (A2102), and Bafilomycin A1(Baf A1, S1413) were from Selleck. The following reagents were obtained from BioLegend: Zombie Aqua Fixable Viability Kit(423102), anti‐mouse CD16/32 antibody(156604), APC/Cyanine7 anti‐mouse CD45 antibody(103116), FITC anti‐mouse CD3 antibody(100204), PerCP/Cyanine5.5 anti‐mouse CD4 antibody(100540), Brilliant Violet 711 anti‐mouse CD8a antibody(100759), Brilliant Violet 421 antihuman/mouse Granzyme B antibody(396414), PE anti‐mouse CD276 antibody (135605), Human TruStain FcX (422302), and PE antihuman CD276 Antibody (351004).

### 4.7 RNA Interference, Plasmids, and Transfection of Cells

siRNAs targeting human WDR12, PES1, BOP1, and CCT7 were purchased from Obio Technology, and the siRNA sequences are as follows:

siWDR12#H1, Forward: GCUGGAAGUGUAGAUUCUATT,
Reverse: UAGAAUCUACACUUCCAGCTT;
siWDR12#H2, Forward: CCACAAAUCGACCAAGAAATT,
Reverse: UUUCUUGGUCGAUUUGUGGTT;
siPES1#H1, Forward: GCCUUGAGAAGAAGAAGUATT,
Reverse: UACUUCUUCUUCUCAAGGCTT;
siPES1#H2, Forward: GCAAGGUCUUCCUGUCCAUTT,
Reverse: AUGGACAGGAAGACCUUGCTT;
siBOP1#H1, Forward: CAGUGAGGAUGAUGACGAATT,
Reverse: UUCGUCAUCAUCCUCACUGTT;
siBOP1#H2, Forward: GGGUGAAUGUAGACCCUGATT,
Reverse: UCAGGGUCUACAUUCACCCTT;
siCCT7#H1, Forward: CGGGAUUACUCAAGGACUAUUTT,
Reverse: AAUAGUCCUUGAGUAAUCCCGTT;
siCCT7#H2, Forward: GGUAUGGAGUAGACAUCAACATT,
Reverse: UGUUGAUGUCUACUCCAUACCTT.

The short hairpin RNA (shRNA) targeting murine Wdr12 was purchased from GenePharma, and its sequences are as follows:

NO.1: 5′‐ccCGAACTAAAGATGGTTCTT‐3′;
NO.2: 5′‐ccTACAGATGAAGAAGATGAA‐3′.

In short, to establish stable melanoma cell lines, the lentiviral expression vector along with packaging plasmids (psPAX2 and pMD2.G) were co‐transfected into HEK293T cells using TurboFect Transfection Reagent (R0531, Thermo Fisher Scientific). After 48 h, the viral supernatant was collected, filtered, and added to melanoma cell cultures, followed by an additional 48‐h incubation. Transduced cells were then selected with 2‐µg/mL puromycin (S250J0, Shanghai BasalMedia Technologies) for 3 days. For transient transfection, siRNA‐mediated knockdown of WDR12, PES1, BOP1, and CCT7 was performed using Lipofectamine RNAiMAX Transfection Reagent (13778150, Thermo Fisher Scientific) according to the manufacturer's instructions.

### 4.8 RNA Extraction and RT–qPCR

Total RNA was extracted from cells using Magzol reagent (R4801, Magen) according to the manufacturer's protocol. One microgram of total RNA was reverse transcribed using the HiScript Q RT SuperMix Kit (R223‐01, Vazyme). Subsequently, qPCR amplification was carried out with SYBR Green qPCR master mix (B21703, Bimake) on a QuantStudio 3 Real‐Time PCR System (Thermo Fisher Scientific). Relative mRNA expression was determined using the ΔΔCt method with GAPDH as the endogenous control. All primer sequences used for qPCR are listed below.

Mouse‐Wdr12‐F: AGGCACGTTTCTACAGCGAG
Mouse‐Wdr12‐R: TAAGGTCCGCAACTTCAGCAG
Mouse‐Gapdh‐F: CATCACTGCCACCCAGAAGACTG
Mouse‐Gapdh‐R: ATGCCAGTGAGCTTCCCGTTCAG
Human‐WDR12‐F: AGCTCCAAACACGCTTCTACA
Human‐WDR12‐R: AGGGCATTCGCAGAAACTGG
Human‐CD276‐F: CTGGCTTTCGTGTGCTGGAGAA
Human‐CD276‐R: GCTGTCAGAGTGTTTCAGAGGC
Human‐CCT7‐F: AGATTGCTGTGACCGTGAAGAAGG
Human‐CCT7‐R: CACTGCATCCACCACCATCTTAGC
Human‐GAPDH‐F: GTCTCCTCTGACTTCAACAGCG
Human‐GAPDH‐R: ACCACCCTGTTGCTGTAGCCAA

### 4.9 Co‐IP and Western Blotting

Total cellular proteins were extracted using a mild lysis buffer supplemented with 1× protease inhibitor and 1× phosphatase inhibitor. The lysate was then incubated overnight at 4°C with Pierce Anti‐DYKDDDDK Magnetic Agarose beads (A36797, Thermo Fisher Scientific). The following day, beads were collected by magnetic separation and washed thoroughly with wash buffer to remove nonspecific binding. Bound proteins were eluted by heating in SDS sample buffer. Equal amounts of protein were separated by 10% SDS‐PAGE and transferred onto PVDF membranes. After blocking with 5% nonfat milk in TBST, membranes were incubated overnight at 4°C with the appropriate primary antibodies. Following washes with TBST, membranes were incubated with HRP‐conjugated goat anti‐rabbit IgG (AS014, Abclonal) or goat anti‐mouse IgG (AS003, Abclonal) secondary antibodies. Protein bands were visualized using an ECL chemiluminescence detection system.

### 4.10 Animal Experiments

All experimental protocols and procedures involving mice were approved by the Institutional Animal Care and Use Committee (IACUC) of Central South University. All C57BL/6 mice (obtained from the Shanghai SLAC Laboratory Animal Center) and NSG mice (obtained from the Shanghai Model Organisms Center) were housed in the animal facilities of Central South University under specific pathogen‐free (SPF) conditions. B16F10 cells with WDR12 knockdown (5 × 10^5^ cells per mouse) were resuspended in 100‐µL PBS and inoculated subcutaneously into the right flank of 6‐week‐old female C57BL/6 mice and NSG mice. One week after inoculation, C57BL/6 mice were randomly grouped and intraperitoneally injected with 100 µg of anti‐PD‐1 antibody or isotype control antibody once every three days. To specifically deplete CD8+ T cells, 200 µg of anti‐CD8α antibody or isotype control antibody was intraperitoneally injected once every three days. To evaluate the in vivo effect of SU14813, wild‐type B16F10 cells were subcutaneously inoculated into female C57BL/6 mice. After random grouping, mice received daily oral administration of vehicle, 10‐mg/kg SU14813, 20‐mg/kg SU14813, or 40‐mg/kg SU14813. To further evaluate the therapeutic effect of combined treatment with SU14813 and anti‐PD‐1 antibody, mice were randomly divided into four groups: (1) vehicle + isotype control antibody, (2) vehicle + anti‐PD‐1 antibody, (3) 10‐mg/kg SU14813 + isotype control antibody, and (4) 10‐mg/kg SU14813+anti‐PD‐1 antibody. To evaluate the efficacy of SU14813 in the anti‐PD‐1 antibody resistant model, mice were randomly divided into three groups: (1) vehicle + isotype control antibody, (2) vehicle + anti‐PD‐1 antibody, and (3) 20‐mg/kg SU14813 + isotype control antibody. Tumor volume and body weight of all mice were measured every 3 days. Tumor volume was calculated according to the following formula: 0.5 × length × width^2^.

### 4.11 Constructing Anti‐PD‐1 Antibody‐Resistant B16F10 Cell Lines

To establish a stable anti‐PD‐1‐resistant melanoma cell line, 5 × 10^5^ B16F10 cells were subcutaneously inoculated into C57BL/6 mice. Mice were intraperitoneally injected with anti‐PD‐1 monoclonal antibody (100 µg per mouse) every three days for a total of three doses. Mice that failed to respond to treatment were selected, and their tumors were aseptically dissected, enzymatically digested, and processed into single‐cell suspensions, which were then cultured in vitro for 7 days. The viable cells were reinoculated into a new cohort of mice, and the above cycle of anti‐PD‐1 treatment, resistant tumor isolation, and in vitro culture was repeated for three rounds. A stable anti‐PD‐1‐resistant cell line was ultimately established and designated as B16F10‐PD1R [50].

### 4.12 Flow Cytometry

For human melanoma cell lines, the flow cytometry analysis steps were as follows: First, dead cells were identified using the Zombie Aqua Fixable Viability Kit, followed by blocking with Human TruStain FcX for 15 min, and then staining with PE‐CD276 antibody for 30 min. For mouse samples, B16F10 tumor tissues were collected, dissected, and mechanically dissociated to prepare single‐cell suspensions, which were then filtered through a 70‐µm cell strainer (352350, Falcon). Dead cells were labeled with the Zombie Aqua Fixable Viability Kit and then blocked with anti‐CD16/32 antibody for 15 min. Subsequently, surface antibody staining was performed for 30 min using the following antibodies: APC/Cyanine7‐CD45, FITC‐CD3, PerCP/Cyanine5.5‐CD4, Brilliant Violet 711‐CD8a and PE‐CD276. For intracellular staining, cells were fixed and permeabilized using the Foxp3/ Transcription Factor Staining Buffer Set (00‐5523‐00, eBioscience), followed by staining with Brilliant Violet 421‐Granzyme B antibody. All samples were detected using FACS LSRFortessa flow cytometer (BD Biosciences), and data were analyzed using FlowJo software (version 10.4).

### 4.13 Immunochemistry and TSA‐Based Multiplex Immunofluorescence (TSA‐mIF)

The paraffin‐embedded tissues of the patients were cut into 4‐µm‐thick sections, baked at 60°C for 120 min, then dewaxed and rehydrated. Heat‐mediated antigen retrieval was performed using EDTA buffer (pH 8.0), and endogenous peroxidase activity was blocked with 3% H_2_O_2_. The sections were then blocked with normal goat serum or 5% BSA for 1 h at room temperature. Subsequently, the sections were incubated overnight at 4°C with the primary antibody (WDR12, 1:3000; CD276, 1:200). The next day, the sections were incubated at room temperature for 1 h with the polymer‐HRP‐labeled goat anti‐mouse/rabbit universal secondary antibody. Immunohistochemical (IHC) staining was performed using DAB, and nuclei were counterstained with hematoxylin. TSA‐mIF staining was performed using a multiplex fluorescence staining kit (Aifang Bio) according to the manufacturer's instructions. Histochemical score (H‐score) assessment was independently performed by two pathologists blinded to the group assignments. For each sample, three random fields of view were captured at 200× magnification. Staining intensity was graded as follows: 0 (none), 1+ (weak), 2+ (moderate), and 3+ (strong). The percentage of positive cells was scored on a scale of 0 to 4: 0 (0%), 1 (1%–25%), 2 (26%–50%), 3 (51%–75%), and 4 (76%–100%). The final H‐score for each section was calculated as the product of the staining intensity score and the proportion score, yielding a range of 0–12. Samples with an H‐score ≤ 6 were classified as the low‐expression group, and those with an H‐score > 6 were classified as the high‐expression group.

### 4.14 Computer‐Assisted Drug Screening

The crystal structure of WDR12 (PDB ID: 6N31 Chain A) was obtained from the RCSB PDB database, and the structure was optimized using the Schrödinger Protein Preparation Wizard. Subsequently, multilevel virtual screening was conducted against the Selleck compound library, including active site identification with SiteMap, ADME/T evaluation with QikProp, and hierarchical molecular docking in the Glide module (HTVS → SP → XP mode; a lower Glide Score indicates more stable binding). Finally, the MM‐GBSA binding free energy of the preferred compounds was calculated (a more negative ΔG_bind value indicates more stable binding), thereby systematically evaluating the binding characteristics of small molecules with the active pocket of WDR12.

### 4.15 BLI

The biotin‐labeled small molecule SU14813 was captured onto SA‐XT biosensors. Unoccupied binding sites on the sensors were then blocked with free biotin to reduce nonspecific background. The sensor‐bound biotin‐SU14813 complex was subsequently incubated with recombinant protein WDR12 (CSB‐EP872413HU, Cusabio). Binding kinetics were then monitored in real time using a Gator Prime system (Gator Bio).

### 4.16 Pull‐Down Assay

SU14813 was conjugated to Sepharose 4B beads (Sangon Biotech, Shanghai) according to the manufacturer's instructions [51]. For short‐term storage, the prepared beads were stored in PBS containing 0.1% sodium azide and stored at 4°C. Cell lysates were incubated with the SU14813‐Sepharose 4B beads (or with DMSO‐Sepharose 4B as a control) in reaction buffer at 4°C overnight with gentle shaking. After incubation, the beads were washed three times with wash buffer. The interaction between SU14813‐Sepharose 4B beads and WDR12 was subsequently analyzed by Western blotting.

### 4.17 Statistical Analysis

Survival analysis was performed using the survival R package (v.3.5.7). Kaplan–Meier survival curves were generated with the survfit function. Univariate Cox regression analysis was applied to estimate the HR for each feature gene. All data are presented as mean ± SEM. Statistical analyses were conducted using GraphPad Prism (version 10.1.2). Group comparisons were carried out using unpaired two‐tailed *t*‐tests and two‐way ANOVA, with a *p*‐value <0.05 considered statistically significant.

## Author Contributions

**Jie Pan**: methodology, investigation, validation, data curation, writing – original draft. **Qian Dong**: resources. **Meng Zhang**: methodology, data curation, writing – original draft, validation. **Juan Su**: conceptualization, data curation, supervision, resources, funding acquisition, writing – review and editing. **Ruimin Chang**: conceptualization, writing – review and editing, data curation, funding acquisition. **Xiang Chen**: supervision, resources, funding acquisition. **Xiaowei Liang**: methodology, writing – review and editing, data curation, investigation. **Yeye Guo**: conceptualization, data curation, supervision, funding acquisition, writing – review and editing. **Mingliang Chen**: data curation. **Lixia Lu**: data curation, funding acquisition. **Guanxiong Zhang**: resources.

## Funding

This work was supported by grants from National Program for High‐Level Medical Talents, National Natural Science Foundation of China (grant No. 82503382; 82403284; 82441043; 82273215), Fundamental and Interdisciplinary Disciplines Breakthrough Plan of the Ministry of Education of China (JYB2025XDXM603), Natural Science Foundation of Hunan Province (2024JJ6648), China Postdoctoral Innovation Talent Supporting Program (BX20230436), and the Fundamental Research Funds for the Central Universities of Central South University.

## Conflicts of Interest

The authors declare no conflicts of interest.

## Supporting information




**Supporting File**: advs76255‐sup‐0001‐SuppMat.docx.

## Data Availability

Bulk transcriptomic data from melanoma patients treated with either anti‐PD‐1 monotherapy or anti‐PD‐1 combined with anti‐CTLA‐4 therapy were obtained from a previously published study by Schadendorf et al. [52]. Raw sequencing data are available in dbGaP under accession number phs000452.v3.p1. Single‐cell RNA‐sequencing data were obtained from the dataset reported by Izar et al. [23], comprising 33 human melanoma tumors, including 15 tumors from patients who did not respond to anti‐PD‐1 therapy. Proteomic data and associated clinical information from patients with stage IV melanoma treated with anti‐PD‐1 therapy or tumor‐infiltrating lymphocytes were obtained from the study by Geiger et al. [53]. Data generated during the current study are available from the corresponding author upon reasonable request.
