# Supplementary material for: Targeting WDR12 Unleashes T‐Cell‐Mediated Antitumor Activity in Melanoma by Destabilizing CD276
Source: Adv Sci (Weinh). 2026 Jun 27;13(52):e76255. doi: 10.1002/advs.76255 (PMC13337114; doi:10.1002/advs.76255)
Supplement: Supplementary file 1 [file ADVS-13-e76255-s001.docx]

**Supplementary Materials**

**FigureS1. Unraveling the dynamic evolution of melanoma and the predictive value of WDR12 for immunotherapy response**

a, The UMAP visualization shows the main cell types present in melanoma tissues. b, The UMAP plot shows the characteristic marker genes of each cell subtype in the single-cell transcriptomics. c, The Dotplot shows the characteristic marker genes of each cell subtype in single-cell transcriptomics. d, The proportion distribution of the six types of melanoma cells in the Naive group and the NR group. e, Univariate Cox regression analysis of the Mela3 characteristic gene in PD-1 blockade therapy based on PFS. f, Univariate Cox regression analysis of the Mela3 characteristic gene in PD-1 blockade therapy based on OS.

**FigureS2. Knockdown of WDR12 promotes CD8+ T cell infiltration and potentiates anti-tumor activity**

a, RT-qPCR and Western blotting analysis of WDR12 expression in B16F10 cells with WDR12 knockdown. b, The CCK8 assay was used to measure the cell viability of B16F10 cells with WDR12 knockdown. c, Representative images of tumor tissue and terminal tumor volume in NSG mice. d, Terminal tumor weight of NSG mice. e-f, C57BL/6 mice were subcutaneously inoculated with negative control (Ctrl) or shWdr12 treated B16F10 cells. (e) Representative flow cytometry gating strategy showing the proportion of CD8+ and CD4+ T cells in CD3 TILs. (f) The proportion of CD8+ T cells in CD3 TILs. g-h, The shWdr12-B16F10 tumor-bearing mice were treated with anti-CD8 antibody or isotype IgG. (g) Monitoring of the mice's body weight during the treatment period. (h) Representative flow cytometry gating strategy showing the proportion of CD8+ T cells in CD3 TILs. All data are presented as mean ± SEM. The p-values were calculated using the unpaired, two-tailed t-test and two-way ANOVA analysis. Not significant (ns), p > 0.05; *p < 0.05; **p < 0.01; ***p < 0.001.

**FigureS3. Knockdown of WDR12 reduces CD276, the level of which correlates with the efficacy of immunotherapy**

a, Proteomics analysis of WDR12 and CD276 expression in the negative control (Ctrl) and shWdr12 tumor tissues. b, Flow cytometry analysis of PD-L1 and CD86 expression in CD45- cells from shWdr12-B16F10 tumors. c, Representative immunohistochemical images showing CD276 expression in patients with different treatment responses. d, Correlation analysis of WDR12 and CD276 expression. e-f, The shWdr12-B16F10 tumor-bearing mice were treated with anti-PD-1 antibody or isotype IgG. (e) Monitoring of the mice's body weight during the treatment period. (f) Representative flow cytometry gating strategy showing GZMB expression in CD8+ T cells. All data are presented as mean ± SEM. The p-values were calculated using the unpaired, two-tailed t-test. Not significant (ns), p > 0.05; *p < 0.05; **p < 0.01; ***p < 0.001.

**FigureS4. WDR12 requires the molecular chaperone CCT7 to stabilize the expression of CD276**

a, Relative intensities of WDR12 and CD276 expression in siNC or siWDR12 melanoma cell lines. b, Western blotting (left) and quantification (right) of WDR12 and CD276 expression in vector control and WDR12-overexpressing melanoma cells. c, Western blotting analysis of CD276 expression in melanoma cell lines transfected with siNC or siPes1/siBop1. d, CD276 expression in melanoma cells treated with CX-5461. e, Relative intensities of SQSTM1/p62 and LC3II expression in siNC or siWDR12 melanoma cell lines. f, Schematic diagram of immunoprecipitation-mass spectrometry (IP-MS) analysis. g, Co-IP confirmed the interaction among CCT7, WDR12, and CD276. h, RT-qPCR analysis of CCT7 and CD276 mRNA expression in melanoma cell lines transfected with siNC or siCCT7. i, Analysis of CD276 expression level by FACS. All data are presented as mean ± SEM. The p-values were calculated using the unpaired, two-tailed t-test. Not significant (ns), p > 0.05; *p < 0.05; **p < 0.01; ***p < 0.001.

**FigureS5. The small molecule compound SU14813 targeting WDR12 down-regulates CD276 and potentiates CD8+ T cell anti-tumor function**

a, Relative intensities of CD276 expression upon treatment with 30 small molecule compounds. b, Quantification of CD276 expression in melanoma cell lines treated with SU14813 by flow cytometry. c, Molecular docking model of SU14813 with WDR12 (3D representation). d, CETSA melting curve of target protein WDR12 in the presence of SU14813. e, Western blotting analysis of WDR12 and CD276 expression after treatment with SU14813. f, Western blotting analysis of WDR12 expression in melanoma cells treated with SU14813 (10 μM) for 48 h, followed by treatment with Baf A1 (400 nM) or MG132 (10 μM) for 4 h. g-l, The B16F10 tumor-bearing mice were treated with SU14813 or vehicle. (g) Representative images of tumor tissues from SU14813-treated mice (n = 5), (h) terminal tumor volume (left) and terminal tumor weight (right). (i) Survival of B16F10 tumor-bearing mice under the indicated treatments. The endpoint was defined as the time required for tumor volume to reach 2,000 mm^3^. (j) Monitoring of the mice's body weight during the treatment period. (k) Plasma biochemical analysis of ALT, AST, CRE, BUN, LDH, and CK in vehicle and SU14813-treated mice. (l) H&E staining analysis of heart, liver, lung, spleen, and kidney tissues from vehicle and SU14813-treated mice. All data are presented as mean ± SEM. The p-values were calculated using the unpaired, two-tailed t-test. Not significant (ns), p > 0.05; *p < 0.05; **p < 0.01; ***p < 0.001. ALT, alanine aminotransferase; AST, aspartate aminotransferase; CRE, creatinine; BUN, blood urea nitrogen; LDH, lactate dehydrogenase; CK, creatine kinase.
